# Versorgung von Parkinson-Patienten in Deutschland: Status quo und Perspektiven im Spiegel des digitalen Wandels

**DOI:** 10.1007/s00115-020-01027-3

**Published:** 2020-11-16

**Authors:** Carsten Eggers, Ingmar Wellach, Sergiu Groppa, Martin Strothjohann, Jochen Klucken

**Affiliations:** 1 Neurologie, Philipps-Universität Marburg, Baldingerstr., 35033 Marburg, Deutschland; 2 Praxis für Neurologie & Psychiatrie Hamburg Walddörfer, Wiesenkamp 22c, 22359 Hamburg, Deutschland; 3 Evangelisches Amalie Sieveking Krankenhaus, Haselkamp 33, 22359 Hamburg, Deutschland; 4 Bewegungsstörungen und Neurostimulation, Klinik und Poliklinik für Neurologie, Forschungszentrum Translationale Neurowissenschaften (FTN) Rhein-Main-Neuro-Zentrum (rmn2), Universitätsmedizin der Johannes Gutenberg-Universität, Langenbeckstr. 1, 55131 Mainz, Deutschland; 5 Medical Park Bad Camberg, Obertorstraße 100–102, 65520 Bad Camberg, Deutschland; 6 Molekulare Neurologie, Universitätsklinikum Erlangen, Schwabachanlage 6, 91054 Erlangen, Deutschland; 7 Fraunhofer IIS, Am Wolfsmantel 33, 91058 Erlangen, Deutschland; 8 Medical Valley Digital Health Application Center GmbH, Promenadestr. 6a, 96047 Bamberg, Deutschland

**Keywords:** Telemedizin, Netzwerke, Digitale Medizin, Integrierte Versorgung, Digitale Gesundheitsanwendung, Telemedicine, Networks, Digital health, Integrated care, Digital health application

## Abstract

Die Parkinson-Krankheit als chronische neurodegenerative Erkrankung bedarf eines engen Zusammenspiels verschiedener Fachdisziplinen, um eine bestmögliche Lebensqualität der Patienten zu gewährleisten. Ein immer wieder identifiziertes Problem ist die insuffiziente Kommunikation zwischen den Protagonisten (z. B. „caregiver“, Ärzte und Therapeuten), insbesondere an den Sektorengrenzen. Die aktuellen Prozess- und Versorgungsketten der Parkinson-Krankheit bilden aber auch aufgrund struktureller Hürden bisher keine gelungene sektorenübergreifende Versorgung ab. Vor dem Hintergrund des neuen Digitale-Versorgung-Gesetzes (DVG) und den damit erstmalig rückfinanzierten, digitalen Gesundheitsanwendungen (DiGAs) können sich nun erstmals innovative, digitale Versorgungs- und Kommunikationsstrukturen etablieren und haben das Potenzial, damit die Versorgung chronischer Erkrankungen, wie z. B. der Parkinson-Krankheit, umfassend zu verändern. Beispiele und Anwendungsszenarien werden in diesem Übersichtsartikel vorgestellt sowie kritisch diskutiert.

## Abstract (en)

As a chronic neurodegenerative disease, Parkinson’s disease requires a close cooperation between different specialist disciplines in order to ensure the best possible quality of life for patients. A problem that has been identified is the inadequate communication between the protagonists (e.g. caregivers, physicians and therapists), especially at the sectoral interfaces. Due to structural hurdles, the current process and supply chains for Parkinson’s disease do not reflect successful cross-sectoral care. Against the background of the new Digital Care Act in Germany that refunds patient-centered digital healthcare applications (DiGA), innovative, digital care and communication structures can now be established and thus comprehensively revolutionize the care of chronic diseases, such as Parkinson’s disease. In this review examples and case application scenarios are presented and critically discussed.

## Hintergrund

Die Parkinson-Krankheit als chronische neurodegenerative Erkrankung bedarf eines engen Zusammenspiels verschiedener Fachdisziplinen und Berufsgruppen, aber auch nichtprofessioneller Beteiligter, um eine bestmögliche Lebensqualität der Patienten zu ermöglichen. Die Betreuung von Parkinson-Patienten in Deutschland ist bisher geprägt durch eine sektorenübergreifende Zusammenarbeit und das komplexe Zusammenspiel der verschiedenen Versorgungspartner (z. B. Ärzte, Therapeuten). Ein immer wieder identifiziertes Problem ist die insuffiziente Kommunikation zwischen den Protagonisten. Das Digitale-Versorgung-Gesetz (DVG) wird mobile Technologien als digitale Gesundheitsanwendungen (DiGAs) zur Versorgungsunterstützung hervorbringen. Anhand dieser Darstellung der zur Verfügung stehenden Prozess- und Versorgungsketten soll die aktuelle Situation (Status quo) und künftige Perspektiven der Parkinson-Versorgung mit einem Fokus auf die Integration digitaler Ansätze aufgezeigt werden.

## Wie ist die Versorgungskette eines Parkinson-Patienten charakterisiert?

Mit Versorgungskette ist im Gesundheitswesen das gliederförmige Ineinandergreifen der verschiedenen Betreuungssektoren gemeint. Diese beinhalten den ambulanten und stationären Sektor (Krankenhaus- und Rehabilitationsbehandlung) unter Einbeziehung der verschiedenen therapeutischen Disziplinen (z. B. aktivierende Therapien). Die Versorgung in der häuslichen Umgebung ist bisher nur auf die Pflege und Hilfsmittelversorgung beschränkt, nur wenige (tele-)medizinische Versorgungsleistungen (z. B. die Videosprechstunde) reichen bis zum Patienten nach Hause. Dieser „häusliche Versorgungssektor“ ist daher noch nicht scharf definiert, wird jedoch durch die Forderung nach Patientenzentriertheit der DiGAs über das DVG eine neue Bedeutung bekommen.

Die ärztliche Betreuung des Parkinson-Patienten erfolgt im ambulanten und stationären Bereich durch Fachärzte für Neurologie, Neurologie und Psychiatrie bzw. Nervenheilkunde (im Folgenden zusammenfassend als „Neurologe“ bezeichnet), wobei der behandelnde neurologisch tätige Facharzt hierbei üblicherweise sektorenspezifische Aufgaben zu erfüllen hat. Zusätzlich übernehmen teils Hausärzte noch den Versorgungsauftrag, wenngleich dies nicht für eine Mehrheit der Parkinson-Patienten zutrifft [30]. Entsprechende Empfehlungen bezüglich der Aufgaben, Indikationsstellungen und Qualitätsmerkmale der verschiedenen Versorgungssektoren wurden bereits von einer Konsensusgruppe publiziert [6]. Eine fachärztliche Spezialisierung auf die Behandlung von Bewegungsstörungen hat sich bisher in Deutschland noch nicht etabliert, obwohl die Leitlinien der Deutschen Gesellschaft für Neurologie (DGN) zumindest für die Diagnosestellung eine besondere Expertise in der klinischen Differenzialdiagnose von Parkinson-Syndromen empfiehlt [1]. Es existiert bundesweit jedoch ein Netzwerk von Praxen mit einem ausgewiesenen Parkinson-Schwerpunkt (s. a. Homepage QUANUP e. V.), das eine überdurchschnittlich hohe Anzahl an Parkinson-Patienten behandelt und in Zusammenarbeit mit der Deutschen Gesellschaft für Parkinson und Bewegungsstörungen (DPG e. V.) und der Deutschen Parkinson Vereinigung (dPV) bei der Entwicklung von Qualitätskriterien mitgewirkt hat. Erste Praxen wurden nach diesen Kriterien seit Anfang dieses Jahres bereits erfolgreich zertifiziert (s. a. Homepage dPV).

Die stationären Leistungen werden in Krankenhäusern aller Versorgungsebenen erbracht, wobei es spezialisierte Parkinson-Fachkliniken (nach Zertifizierungskriterien der dPV) sowie hochspezialisierte Schwerpunktzentren, meist in den Universitätskliniken, gibt. Als neues Versorgungsangebot haben sich seit kurzer Zeit Parkinson-Tageskliniken etabliert. Diese teilstationäre Versorgung bietet den Vorteil einer tagesklinischen Behandlung, ist jedoch abhängig von individuellen Vertragsverhandlungen mit den Kostenträgern.

**Figure 1:**
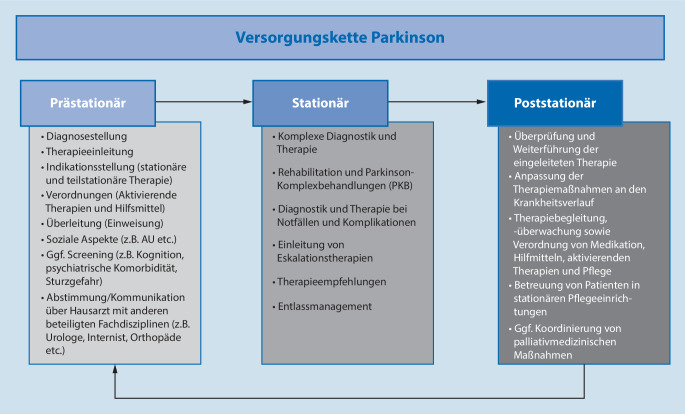

Im Folgenden beschreiben wir die verschiedenen Aufgaben der Versorgungsstationen (Abb. 1):

1. *Ambulanter Bereich (prästationär):* Zu Beginn der Betreuung stehen neben der Diagnosestellung präventive Maßnahmen, die Therapieeinleitung sowie die Koordination und Überwachung der Therapie. Erster Übergang in der Versorgungskette ist dann die Indikationsstellung für eine stationär geführte Behandlung.
  Zu den formalen Aufgaben der versorgenden Ärzte beim Übergang des Patienten in den jeweils anderen Versorgungssektor gehört auch eine Überleitungsdokumentation.
2. *Stationärer Bereich:* Im stationären Bereich werden in Abhängigkeit von der Fragestellung entsprechende Maßnahmen eingeleitet (rehabilitative oder akute Behandlung). Der Klinikarzt wird auf der Kenntnis der aktuell durchgeführten Therapie entsprechende diagnostische und therapeutische Maßnahmen einleiten und dementsprechend die Therapie anpassen. Der Behandlungsverlauf (Epikrise) sowie die Therapieempfehlungen werden am Ende des Aufenthaltes zusammengefasst und im Rahmen eines Entlassbriefes mitgeteilt.
3. *Ambulanter Bereich (poststationär):* Der niedergelassene Neurologe hat im Sinne einer „Rückübernahme“ des Patienten wiederum die Aufgabe, die Epikrise des Patienten zu würdigen und die Verordnungen sowie den Medikamentenplan zu überprüfen. Sinn und Zweck dieser Aufgabe ist die Weiterführung der Behandlung und Überprüfung der Praktikabilität, der Verträglichkeit sowie des Nutzens der verordneten Therapie (auch wirtschaftlich) aus der langjährigen Kenntnis des Patienten und seiner sozialen Bezüge. In der Folge verordnet der niedergelassene Neurologe die Therapien und verantwortet diese medizinisch gegenüber dem Patienten und wirtschaftlich gegenüber den Kostenträgern. Zu seinen weiteren Aufgaben gehört die Koordination der ambulanten Therapie sowie die Indikation und Verordnung von Heil- und Hilfsmitteln sowie ambulanter Pflege.
4. Am Ende der Versorgungskette ist die Versorgung von Patienten in *stationären Pflegeeinrichtungen* möglich, idealerweise unter Einbeziehung und Koordinierung palliativmedizinischer Maßnahmen (in der Regel durch den niedergelassenen Neurologen in Zusammenarbeit mit einem Palliativmediziner und einem Palliative-care-Team).

Einen schematischen Überblick über den Ablauf der Versorgungskette gibt Abb. 1.

Eine Besonderheit der Parkinson-Erkrankung ist die komplexe Symptomkombination mit zahlreichen motorischen und nichtmotorischen Störungen, die insbesondere in späteren Krankheitsstadien in der Regel ein multimodales Therapieregime erfordern sowie ein eher geriatrisch geprägtes Patientenklientel mit einer zum Teil erheblichen Komorbidität. Dabei ist sowohl von einer Zunahme der Parkinson-Patienten allgemein als auch von deren Multimorbidität auszugehen [6]. Daher müssen sowohl im ambulanten als auch im stationären Bereich alle in Anspruch genommenen therapeutischen Disziplinen von den Behandlungsabläufen und den Therapiemodifikationen umfassend informiert werden. Dies erfordert eine interdisziplinär gut koordinierte Strategie inklusive entsprechenden Kommunikationsstrukturen, was im Alltag eine gewisse Herausforderung für denjenigen darstellt, der die ambulante Versorgung im Wesentlichen koordiniert. Durch die reibungslose Interaktion innerhalb der Versorgungskette kann das Management der Erkrankung im zeitlichen Verlauf und durch die verschiedenen Stadien der Erkrankung optimiert werden.

Es besteht allerdings in Deutschland weitgehend Konsens darüber, dass immer noch nicht die individuell optimale Behandlung für jeden einzelnen Erkrankten durchgehend gewährleistet ist [9]. Insbesondere die Medikation ist eine große Herausforderung – und diese kann als „Eckpfeiler“ im Management der Parkinson-Erkrankung angesehen werden. Gerade hinsichtlich der Medikation sind Parkinson-Patienten als „Risikopatienten“ einzuschätzen, weil sehr häufig mehr als drei Arzneimittel verordnet werden, Complianceprobleme auftreten und in der Regel interaktions- oder nebenwirkungsträchtige Arzneimittel eingenommen werden [15, 23].

Andere Daten belegen jedoch, dass die Medikation insbesondere beim Übergang zwischen ambulanter und stationärer Therapie gewollt oder ungewollt verändert wird. Dies kann zum Teil erhebliche Ausmaße annehmen. So wurden nach diesen Daten nicht nur etwa ein Drittel der ambulant verordneten Medikamente abgesetzt, sondern andererseits nach Entlassung nur gut 60 % der stationär verordneten Medikamente weitergeführt [2, 14]. Jedoch auch durch Fehler in der Informationskette am Übergang der Versorgungssektoren kommt es häufig zu akzidentellen Veränderungen in der medikamentösen Versorgung. Insgesamt ist es naheliegend, dass Schnittstellenprobleme insbesondere bezüglich der medikamentösen Therapie zu erheblichen medizinischen und gesundheitsökonomischen Konsequenzen führen [7, 20, 26]. Hierdurch kommt es alarmierend häufig zu einer Verschlechterung der gesundheitlichen Situation der Betroffenen und konsekutiv zu erneuten Krankenhausaufnahmen [29].

## Künftige Lösungsansätze für eine optimierte Parkinson-Versorgung

Die zu erwartenden Veränderungen ausgelöst durch das DVG [3] haben das Potenzial, patientenzentriert wirkende und damit transsektorale, digitale Gesundheitsanwendungen („DiGAs“) in eine Kostenerstattung zu bringen. Dadurch wird die Rolle des Versorgungsmehrwertes für den Patienten in den Vordergrund gehoben und die resultierenden Gesundheitsdienstleistungen – DiGAs – können gerade interdisziplinäre und sektorenübergreifende Versorgungskonzepte nachhaltig unterstützen. Ein wichtiger Mehrwert wird dabei die digital unterstützte (tele-)medizinische Versorgung der Patienten sein. Im Gegensatz zu einer Vielzahl bereits etablierter telemedizinischer Anwendungen – wie z. B. dem Telekonsil zwischen Ärzten unterschiedlicher Fachgruppen oder Expertisen oder auch der Telesprechstunde – wird dabei speziell die patientenzentrierte Anwendung im Vordergrund stehen. Für die DiGAs sind die sog. „positiven Versorgungseffekte“ wissenschaftlich nachzuweisen, die nicht nur den medizinischen Nutzen adressieren, sondern sogar nur eine strukturelle oder prozedurale Verbesserung erzielen müssen (wie z. B. der bessere Zugang zur medizinischen Versorgung oder die Verbesserung der Versorgung nach Standards oder Leitlinien). Von Letzterem werden v. a. integrierte Versorgungskonzepte profitieren, die eben nicht nur die Krankheit und die Symptomausprägung verbessern, sondern auch eine Vielzahl struktureller und prozeduraler Aspekte der Versorgungskette. Die digitale Versorgungsunterstützung wird durch Smartphone-Applikationen, körpernahe Sensoren und lernende Algorithmen der „künstlichen Intelligenz“ geleistet werden, die auf die nun endlich absehbaren digitalen Strukturen wie der Telematikinfrastruktur (https://www.gematik.de/telematikinfrastruktur/) und den elektronischen Patientenakten (EPAs) aufsetzen werden. Ein interessanter Aspekt für die Start-ups und Firmen, die solche DiGAs anbieten werden, ist v. a. die im DVG verankerte Rückfinanzierung – „die Digitale Gesundheits-APP auf Rezept“, die bereits während der wissenschaftlichen Evaluation der positiven Versorgungseffekte erfolgen kann („Fast-Track“). Durch diesen neuen Markt werden die bisherigen Hürden (Medizin-Produkt Verordnung, MDR; Europäische Datenschutzgrundverordnung, DSGVO) lediglich zum Kostenfaktor, da nach erfolgreicher Lösung dieser Anforderungen belastbare Geschäftsmodelle möglich werden. Gleichzeitig bedeutet das auch, dass die ersten DiGAs für Parkinson-Patienten sicherlich in den nächsten 12 Monaten zu erwarten sind und auch die etablierten Versorgungsketten ergänzen werden.

## Telemedizinische Kommunikationsplattformen für besseres Patientenmanagement

Telemedizinisch unterstützte Versorgungskonzepte basieren grundsätzlich auf der Bereitstellung medizinischer Information für die Behandlung des Patienten. Diese Information kann zwischen den klassischen, sektoralen Versorgern ausgetauscht werden (Krankenhaus, Neurologe/Bewegungsstörungsspezialist, Hausarzt) oder direkt mit dem Patienten in seiner häuslichen Umgebung (Videovisite, technologieunterstütztes Telemonitoring, spezifisch zugeschnittene Informationen über Krankheitscharakteristika, Behandlungskonzepte oder Nebenwirkungen). Vor allem von der direkten Einbindung des Patienten verspricht man sich eine optimierte, auf den individuellen Patienten zugeschnittene Behandlung und Einbindung des Patienten in den Versorgungsprozess, die auch von neuen Verfahren der künstlichen Intelligenz anhand der bereitgestellten medizinischen Daten unterstützt werden: „tailored/precision medicine“. Es leuchtet daher ein, dass der zentrale Baustein einer optimierten Parkinson-Versorgung eine verbesserte Kommunikation dieser Informationen zwischen allen Leistungserbringern und dem Patienten selber ist. Grundsätzlich ist die bessere Vorhaltung und Verfügbarkeit notwendiger Informationen für alle Akteure eine Stärke der Digitalisierung. Wenn hier die wichtigen, grundlegenden Fragen der Sicherheit, Interoperabilität und insbesondere der Selbstbestimmung des Patienten gelöst sind, ist nicht nur eine enorme Effizienzsteigerung seitens der Leistungserbringer abzusehen, sondern auch ein erheblicher Mehrwert für den Patienten ableitbar. Neue IT-gestützte Kommunikationsplattformen sind ein Instrument der zunehmenden Digitalisierung der Medizin, von der Parkinson-Patienten grundsätzlich profitieren können, da sie in der Regel mit einer Vielzahl an unterschiedlichen medizinischen Berufsgruppen aufgrund ihrer Erkrankung interagieren. Der Patient begibt sich mit der ersten Diagnose auf eine Reise durch die vor ihm liegenden Krankheitsphasen („patient journey“; [18]). Aufgrund des chronischen Verlaufes stellt das Parkinson-Syndrom dabei durch die Notwendigkeit zur Langzeitversorgung eine besondere Herausforderung an die Leistungserbringer dar. Gerade aus Sicht der Technologie, die den chronischen Patienten zukünftig in der häuslichen Umgebung begleiten und unterstützen wird, ist im Gegensatz zu akuten Erkrankungen, bei der die einzeitige Behandlung beim niedergelassenen Arzt oder im Krankenhaus im Vordergrund steht, eine andere Dimension der Kommunikation zwischen dem Patienten, seinem betreuenden Arzt bzw. der unterstützenden Technologie notwendig [16]. Aus der Abfolge der einzelnen Behandlungen wird eine langfristige Begleitung des Patienten. Der Informationsaustausch zwischen diesen Akteuren funktioniert derzeit unzureichend. Er ist kaum harmonisiert und für den Patienten in der Regel nur schwer durchschaubar. Neue, daten-/informationsgetriebene und über moderne IT-Plattformen unterstützte Patientenmanagementkonzepte können in Zukunft möglicherweise patientenzentriert den Kommunikationsprozess unterstützen und entsprechend effizienter und v. a. zeitgerechter anbieten, als die derzeitige klassisch-sektorale Versorgungsstruktur dies ermöglichen kann. Implementiert sind diese Modelle jedoch bisher noch nicht. Der Patient – genauso wie die ihm nahestehenden pflegenden Angehörigen – profitieren dann vom individualisierten und geleiteten Versorgungsmanagement. Die krankheits- und gesundheitsrelevanten Daten können so für alle Akteure zur Versorgung des Patienten bereitgestellt werden. Die Organisation der Versorgung wäre damit um die Bedürfnisse des Patienten herum sektorenübergreifend digitalisiert: Rezepte, Behandlungsdokumente ambulant wie stationär, häusliche und kontinuierliche Diagnostik- und Therapieprogramme werden dann vereinheitlicht und dadurch optimal entlang der Patienten-Journey nutzbar sein. Durch die Entwicklung neuer tragbarer Sensoren können Symptome schneller erkannt und objektiv vermessen werden. Dadurch kann die Versorgung proaktiv und frühzeitig unterstützt werden und interdisziplinäre Behandlungsteams können vergleichbare und strukturierte Behandlungsinformationen einfacher und einheitlicher austauschen. Dies hat das Potenzial, gemeinsame Behandlungsziele zu definieren und mit dem Patienten und den Angehörigen zusammen die integrierten Versorgungskonzepte optimal zu unterstützen.

Einheitliche elektronische Patientenakten (EPA) und IT-Infrastrukturen (wie die Telematikinfrastruktur) können dabei als dringend benötigtes Infrastrukturmodul diese optimierte Informationsbereitstellung unterstützen.

## Bisherige telemedizinische Dienstleistungen für Parkinson-Patienten

Als erste telemedizinische Anwendung in der Regelversorgung ist hier die Onlinevideosprechstunde zu nennen, die seit 2017 auch für die ärztliche Betreuung von Parkinson-Patienten als telemedizinische Leistung zu nutzen ist [4]. Der Neurologe kann dabei einen zertifizierten Videodienstanbieter auswählen und über ein Videosystem bestehend aus Bildschirm, Kamera, Mikrofon und Lautsprecher mit dem Patienten über eine Internetverbindung in seiner häuslichen Umgebung kommunizieren. Die Vergütungsstrukturen dieser Leistung und auch die Erfahrungen und Bewertungen des Nutzens stecken jedoch noch in den Kinderschuhen, sodass das Interesse sowohl bei Ärzten als auch Patienten noch sehr verhalten ist, obwohl das Interesse an telemedizinischen Behandlungsformen in der Bevölkerung groß ist (https://www.bitkom.org/sites/default/files/file/import/Bitkom-Pressekonferenz-Digital-Health-15-09-2016-Praesentation-final.pdf). Die Videosprechstunde wird im neuen Digitale-Versorgung-Gesetz (DVG) daher auch explizit als neue Versorgungsoption aufgegriffen.

Neben der allgemeinen Onlinevideosprechstunde wird in Deutschland die ambulante videounterstützte Therapie von Parkinson-Patienten zwar noch nicht im Rahmen der Regelversorgung, aber in Selektivverträgen mit den Krankenkassen (§ 140 a SGB V – integrierte Versorgung) unterstützt [11, 28], wobei einige Verträge mit den Kostenträgern aktuell nicht weiter verlängert wurden. Die wissenschaftliche Evaluation dieses Angebotes ist bisher leider nur gering, obwohl die Anwendung in der klinischen Erfahrung durchaus positiv zu werten ist [22]. Ähnliche videobasierte Telemedizinkonzepte werden in Deutschland auch vom Bayerischen Staatsministerium für Gesundheit und Pflege unterstützt: „Telemedizinische Live-Betreuung von Parkinson-Patienten“ (www.stmgp.bayern.de/telemedizin/telemedizinische-live-betreuung-von-parkinsonpatienten-in-der-haeuslichen-umgebung-durch-bilaterale-livestream-video-beobachtung). Die Idee, aus dem häuslichen Bereich bzw. Alltag des Patienten Videoaufnahmen zu nutzen, um die vornehmlich motorischen Einschränkungen zu erfassen und dem Arzt zu visualisieren, ist mit dem Einzug der tragbaren Kameras und Smartphones entstanden [13]. In Schweden werden videobasierte Home-Monitoring-Konzepte zur Überwachung der kontinuierlichen dopaminergen Therapie mittels Pumpenapplikationen evaluiert [33]. Auch die Kombination von Videoanalyse und tragbaren Sensoren stellt eine interessante Entwicklung zur kontinuierlichen häuslichen Betreuung der Parkinson-Patienten dar [27]. Ein ähnliches telemedizinisches Versorgungskonzept spezifiziert auf die Behandlung von Parkinson-Patienten an der Universität Rochester, USA konnte zeigen, dass die Versorgung der Patienten ähnlich gut ist wie der Ambulanzbesuch beim Neurologen [20, 21].

## Mobile Technologien und sensorbasierte Monitoringkonzepte

Durch das DVG unterstützt, werden sich diverse patientenzentrierte Technologieentwicklungen vom Prototyp zu DiGAs und ähnlichen Gesundheitsanwendungen weiterentwickeln. Mobile und tragbare Sensoren werden in Smartphone-basierte Anwendungen integriert werden und den Nachweis des gesetzlich geforderten „positiven Versorgungseffektes“ anstreben. Diese „wearables“ – also tragbare Sensordiagnosesysteme – wurden innerhalb der letzten Jahre zunehmend erforscht und finden schon schrittweise Anwendung bei Parkinson-Patienten [8, 16, 17, 24]. In einem Versorgungskonzept liefern diese Sensoren neue objektive Parameter der unterschiedlichen Symptomausprägungen [12] und werden speziell die häusliche Versorgung unterstützen [25]. Die sensorbasierten, mobilen Systeme werden derzeit noch hauptsächlich in klinischen Studien als objektive Zielparameter verwendet [5], haben jedoch auch das Potenzial, in der klinischen Versorgung die Behandlung zu verändern. Durch die Tragbarkeit lassen sich insbesondere Home-Monitoring-Anwendungen verwirklichen, die sowohl in der Behandlung als auch in klinischen Studien als sog. „real life targets/outcomes“ zunehmend Verwendung finden. Eine nutzenorientierte, einheitliche Darstellung der relevanten Parameter muss dann für den Patienten und seine Angehörigen verständlich aufbereitet und dargestellt werden. Bei klinischen Studien können diese sensorbasierten Parameter direkt als Zielvariablen genutzt werden, während sich diese Parameter jedoch inhaltlich in das Management der weiteren patientenspezifischen und krankheitsrelevanten Informationen (wie Symptome, Diagnosen, Therapieformen, Wirkung und Nebenwirkung, Monitoringoptionen oder Pflegenotwendigkeiten) für die tägliche Versorgung noch konkreter eingliedern müssen. Dieses „Precision-medicine“-Konzept funktioniert dann, wenn die entsprechenden Informationen jederzeit abrufbar sind und allen beteiligten Akteuren (Arzt, Therapeut, Patient, Angehöriger, Apotheker, Krankenkasse etc.) in geeigneter (und regulierter) Form zur Verfügung stehen.

Als Beispiel für die Zielsymptome beim Parkinson-Syndrom, die durch Sensoren erfasst werden können, sind insbesondere die Gangstörungen zu nennen. Sie limitieren die Lebensqualität und Mobilität des Patienten, sind Ziel diverser medikamentöser und nichtmedikamentöser Therapieansätze und bestimmen im Krankheitsverlauf durch die damit verbundenen Komplikationen (Freezing, Stürze, Verlust der Gehfähigkeit) einen erheblichen Anteil der Komorbiditäten und Gesundheitskosten. Die Versorgungsforschung zur Überprüfung, ob diese neuen Technologien tatsächlich auch einen Mehrwert in der Behandlung erzielen können, wird die nächsten Jahre unter dem Stichwort „digital health“ beherrschen und die telemedizinische Versorgung von Parkinson-Patienten zunehmend unterstützen. Hierbei geht es vor allem um einen Transfer von Digital-health-Anwendungen in den Versorgungsalltag. Dadurch wird sich auch die Art und Weise, wie die Gesundheitsversorgung in Deutschland umgesetzt wird, erheblich verändern. Allem voran wird sich jedoch die Rolle aller Gesundheitsdienstleister in der Kommunikation untereinander, aber auch mit dem Patienten als operativer Teil des Behandlungsteams ganz im Sinne integrierter Versorgungskonzepte in der nahen Zukunft rapide wandeln.

## Wie können digitale Lösungen in die existierende Versorgungsstruktur integriert werden?

Die Interaktion der verschiedenen Versorgungspartner (z. B. Hausarzt, Neurologe, Therapeuten, Pflegedienst, Sozialarbeiter, Parkinson-Nurse, Parkinson-Assistenten [PASS] etc.) erfordert ein enges Zusammenspiel dieser Akteure. Das Stichwort „integrierte Versorgung“ stellt in diesem Kontext die Integration der verschiedenen Disziplinen z. B. zu optimierter Risikoanalyse, Langzeitversorgung oder Selbstmanagement dar [19].

Integrierte Versorgungsmodelle mit Ansätzen zur Netzwerkbildung sind international noch nicht stark ausgeprägt. Eine Vorreiterrolle hat in diesem Kontext schon früh das Parkinson-Net in Nijmegen, Niederlande (https://www.parkinsonnet.com) gespielt: Hier wurde gezeigt, dass eine netzwerkbasierte Versorgung von Parkinson-Patienten z. B. zu einer Verbesserung der Lebensqualität führen kann [32]. In Kanada ist die Versorgungsdichte ein großes Thema: Hier wurde im Staat Ontario ebenfalls ein integrierter Versorgungsansatz im Rahmen einer randomisierten, kontrollierten Studie untersucht, auch hier konnte durch den interdisziplinären, koordinativen Einsatz von Versorgungsstrukturen eine deutliche Verbesserung der Lebensqualität erreicht werden [31]. In Deutschland waren lange keine Bemühungen zu diesem Thema erkennbar. In den letzten Jahren wurden nun verschiedene integrierte, netzwerkbasierte Versorgungsstrukturen geschaffen, die vor allem auf standardisierten, strukturierten Kommunikationsprozessen aufbauen. Unterschiedlich gelöst wurden die Projekte in Modellregionen in Deutschland. Zu nennen sind hier z. B. das Düsseldorfer Visitenmodell, das Kölner Parkinson Netzwerk mit Hausbesuchen einer Parkinson-Nurse [10], die Qualitätsoffensive der Berliner Parkinson-Schwerpunktpraxen mit Qualitätszirkeln, gemeinsamen Schulungsmaßnahmen (www.parkinsonverein.de) oder jüngst das Parkinsonnetz Münsterland+ (Zusammenschluss regional beteiligter Versorgungspartner für Vernetzung und Austausch, Bündelung von Expertise) oder das Parkinson-Netzwerk Ost-Sachsen (PANOS; Vernetzung ambulante/stationäre Strukturen, Nutzung Telemedizin, Case-Management etc.). In Hessen hat sich aktuell die Parkinson Netzwerk Allianz Marburg (PANAMA; http://www.ukgm.de/ugm_2/deu/umr_neu/16242.html) unter Federführung der Klinik für Neurologie am Universitätsklinikum Marburg mit einem Fokus auf digitale Interaktion, u. a. im Rahmen einer elektronischen Visite, gebildet. In Hamburg hat sich ein sektorenübergreifend ambulant-stationär verzahntes lokales Versorgungsnetz am Amalie Sieveking-Krankenhaus formiert. Diese unterschiedlichen integrierten Versorgungsansätze haben gemeinsam, dass (meistens) die unterschiedlichen Akteure der Versorgungskette verzahnt werden, um besser miteinander zu interagieren. Fehlend sind in diesen Modellprojekten meist bisher noch die Umsetzung digitaler Versorgungsprozesse, um eine nahtlose Interaktion und somit einen bestmöglichen Nutzen für Patienten und Angehörige zu gewährleisten sowie entsprechend angepasste Vergütungsmodelle seitens der Kostenträger. Gründe für die mangelnde Implementierung digitaler Strukturen sind vor allem mangelnde (finanzielle) Ressourcen oder Erstattungskonzepte, der Fokus auf persönlichen Austausch oder die Komplexität von Systemlösungen. Zudem stellen die aufwendigen datenschutzrechtlichen Abstimmungsprozesse zwischen Gesundheitsdienstleistern, Krankenkassen, Verbänden etc. oftmals eine wesentliche Hürde dar. Diesen Faktoren wird nun jedoch u. a. in Modellprojekten in Münster oder Sachsen durch die Errichtung digitaler Behandlungspfade Rechnung getragen.

## Kritische Wertung der digitalen Technologien

Die hier vorgestellten Konzepte und Ideen sind z. T. bereits in der Praxis etabliert (z. B. Telemedizin mittels digitaler Sprechstunden, Videotherapie), z. T. jedoch erst in der Entwicklung. Bisher fehlt bei vielen der digitalen Konzepte eine klare Kosten-Nutzen-Bewertung, die insbesondere die Akzeptanz, Adhärenz oder Compliance auf Patientenseite untersucht hat. Neben den teils hohen Initialkosten existieren ebenso bisher keine klaren Belege für Kostenreduktionen. Somit ist insbesondere die wissenschaftliche Evaluation der DiGAs aus unserer Sicht ein Kernkriterium für den mittel- und langfristigen Erfolg der neuen Technologien.

Die Wirkung und der Einfluss von Digitalisierungsprozessen auf chronisch kranke, multimorbide und/oder ältere Patientengruppen (wie z. B. Parkinson-Patienten) sind bisher nicht ausreichend untersucht worden. Insbesondere fehlen Daten zu potenziell negativen Aspekten wie z. B. Überforderungserleben, Stress oder Sorge vor der korrekten Bedienung. Auch die Kompetenzen zur Bedienung oder Umsetzung der digitalen Technologien im Alltag sind teils evtl. fehlend und müssen geschult werden. Hier sind vor allem Aspekte wie Selbstmanagement oder Technologiekompetenz in den Fokus zu nehmen. Um ein besseres Verständnis von Patientenwünschen und Anforderungen an neue Technologien zu erhalten, werden z. B. in innovativen Versorgungsforschungsprojekten Fokusgruppen durchgeführt, um maßgeschneiderte Ansätze zu entwickeln (z. B. www.icare-pd.ca). Die Technikaffinität bei älteren, multimorbiden Patienten ist nach eigenen Erfahrungen der Autoren als insgesamt sehr divers einzustufen und muss bei den Implementierungsansätzen Berücksichtigung finden.

Zu diskutieren bleibt weiterhin, dass viele Patienten nach hochspezialisierter Versorgung in der stationären bzw. häuslichen Pflege versorgt werden und die digitalen Anwendungen (z. B. Onlinesprechstunde) überhaupt einen Zugang zum Versorgungssystem ermöglichen.

## Ausblick und Fazit für die Praxis

Das deutsche Gesundheitssystem durchlebt aktuell einen disruptiven Wandel zu einer stärker ausgeprägten Digitalisierung von gesundheitsbezogenen Dienstleistungen sowie Kommunikationsprozessen. Die COVID-19-Pandemie hat in Teilbereichen diese Entwicklung noch beschleunigt. Insbesondere durch die rasche Umsetzung telemedizinischer Versorgungskonzepte (v. a. Videosprechstunden entsprechend den Vorgaben der Bundesärztekammer) konnten neu entstandene Versorgungslücken geschlossen werden. Diese Digitalisierungsmaßnahmen folgten jedoch oft dem Engagement einzelner Protagonisten und keinen strukturellen Vorgaben.

Die digitalen Möglichkeiten, v. a. aber die Onlinesprechstunde, waren für viele in der klinischen Versorgung Tätige, vor allem im niedergelassene Bereich, aus medizinischen, aber auch wirtschaftlichen Gründen elementar: Es konnten positiv getestete Parkinson-Patienten online weiter betreut werden, unnötige Belastungen der Patienten durch Wegstrecken mit Infektionsrisiko wurden vermindert etc. Einzelne Kassenärztliche Vereinigungen haben dafür vorübergehend ermöglicht, Onlinesprechstunden, aber auch Telefonsprechstunden entsprechend abzurechnen. Diese Regelung wurde aber nach Besserung der Situation wieder zurückgenommen. Somit bleibt abzuwarten, wie sich die Systemimplementierung digitaler Technologien beim Fortgang der Pandemie entwickelt.

Die Parkinson-Krankheit als chronische neurodegenerative Erkrankung nimmt hier durch die Notwendigkeit der sektorenübergreifenden Versorgung sowie des langfristigen Versorgungsbedarfs bei einer komplexen Symptomatik sicherlich eine exemplarische Rolle auch für andere chronische Erkrankungen ein. Die deutsche Versorgungslandschaft Parkinson ist durch die DPG in Form der Arbeitsgruppen „Netzwerke & Versorgung“ sowie „Telehealth-Services und Gesundheitstechnologien“ gut aufgestellt, diese Themen künftig sowohl gesundheitspolitisch als auch wissenschaftlich zu begleiten, zu moderieren und neue Impulse zu setzen, um die Versorgung von Parkinson-Patienten auch zukünftig federführend mitzugestalten.
